# Supplementary figures and images for: Swab pooling: A new method for large-scale RT-qPCR screening of SARS-CoV-2 avoiding sample dilution
Source: PLoS One. 2021 Feb 4;16(2):e0246544. doi: 10.1371/journal.pone.0246544 (PMC7861376; doi:10.1371/journal.pone.0246544)

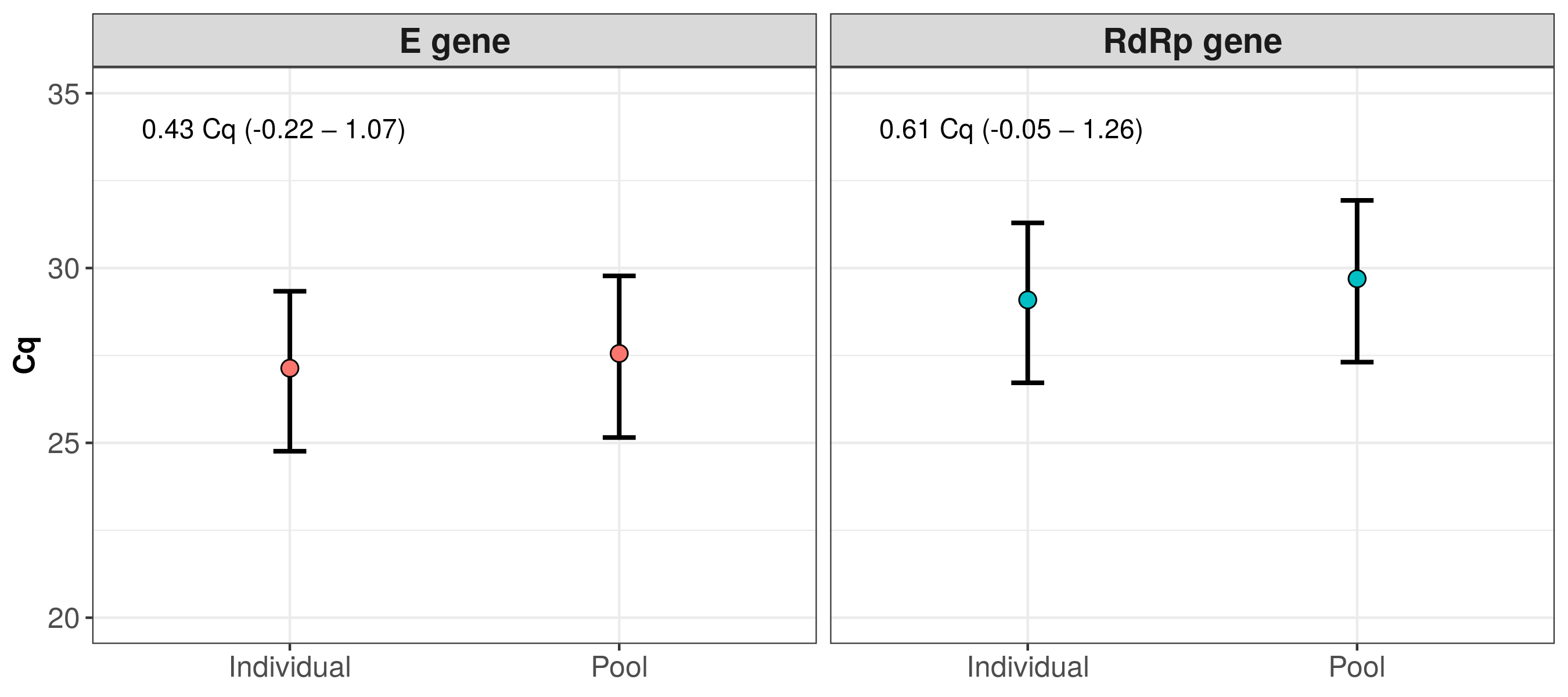

Supplement: S1 Data — (GZ) [file pone.0246544.s001.gz › output/img/cq_differences_dilution.png]

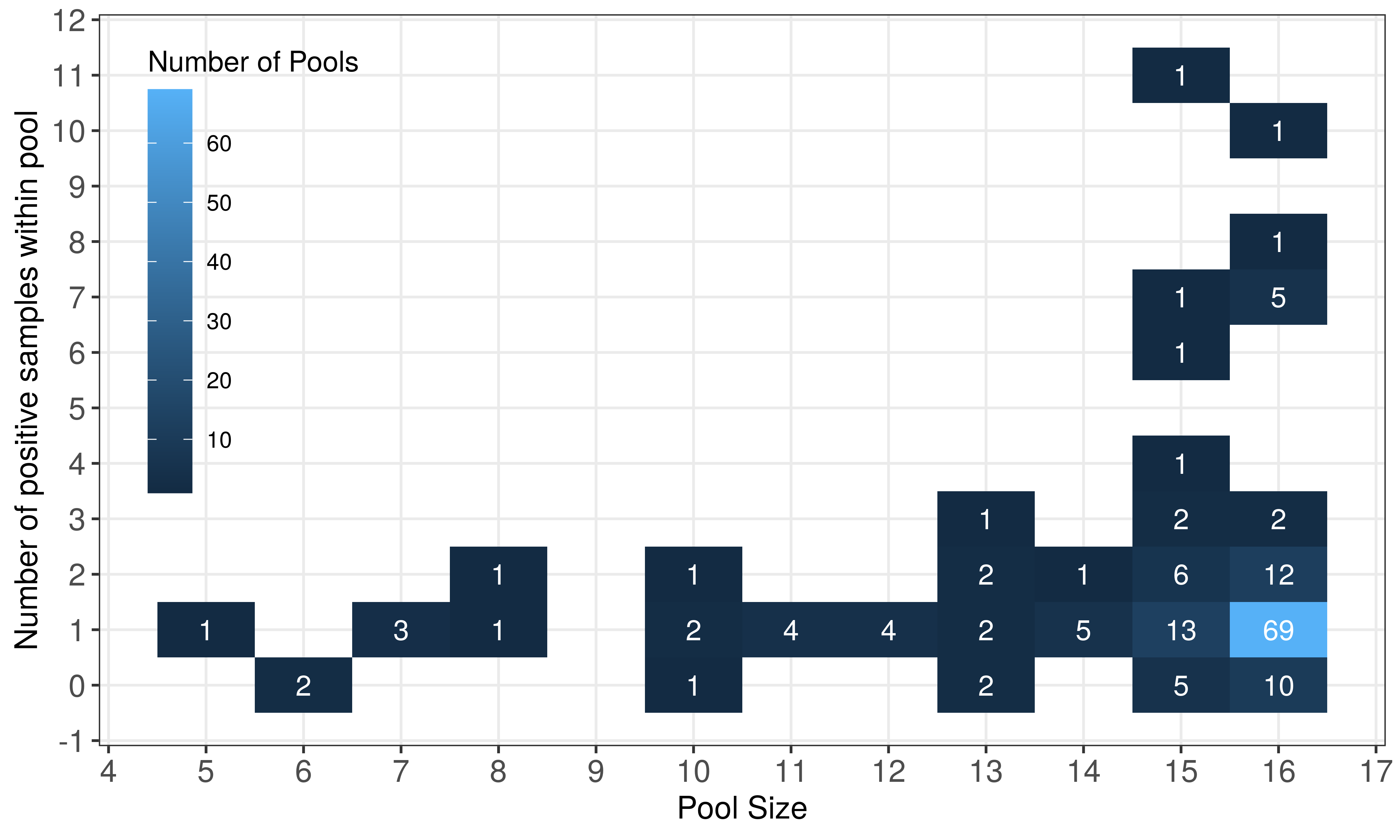

Supplement: S1 Data — (GZ) [file pone.0246544.s001.gz › output/img/pool_size_vs_n_pos_vs_n_pools.png]

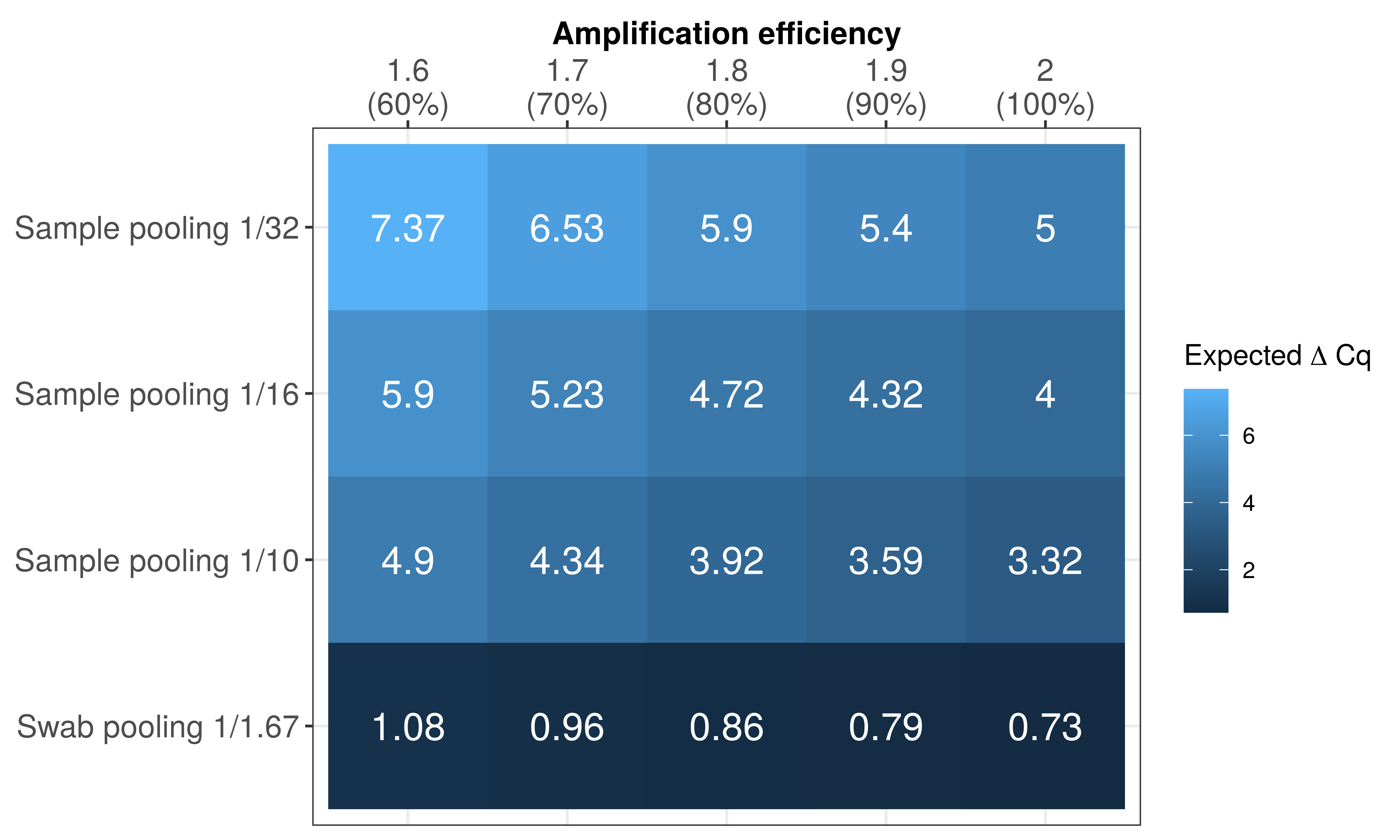

Supplement: S1 Data — (GZ) [file pone.0246544.s001.gz › output/img/theoretical_differences.png]

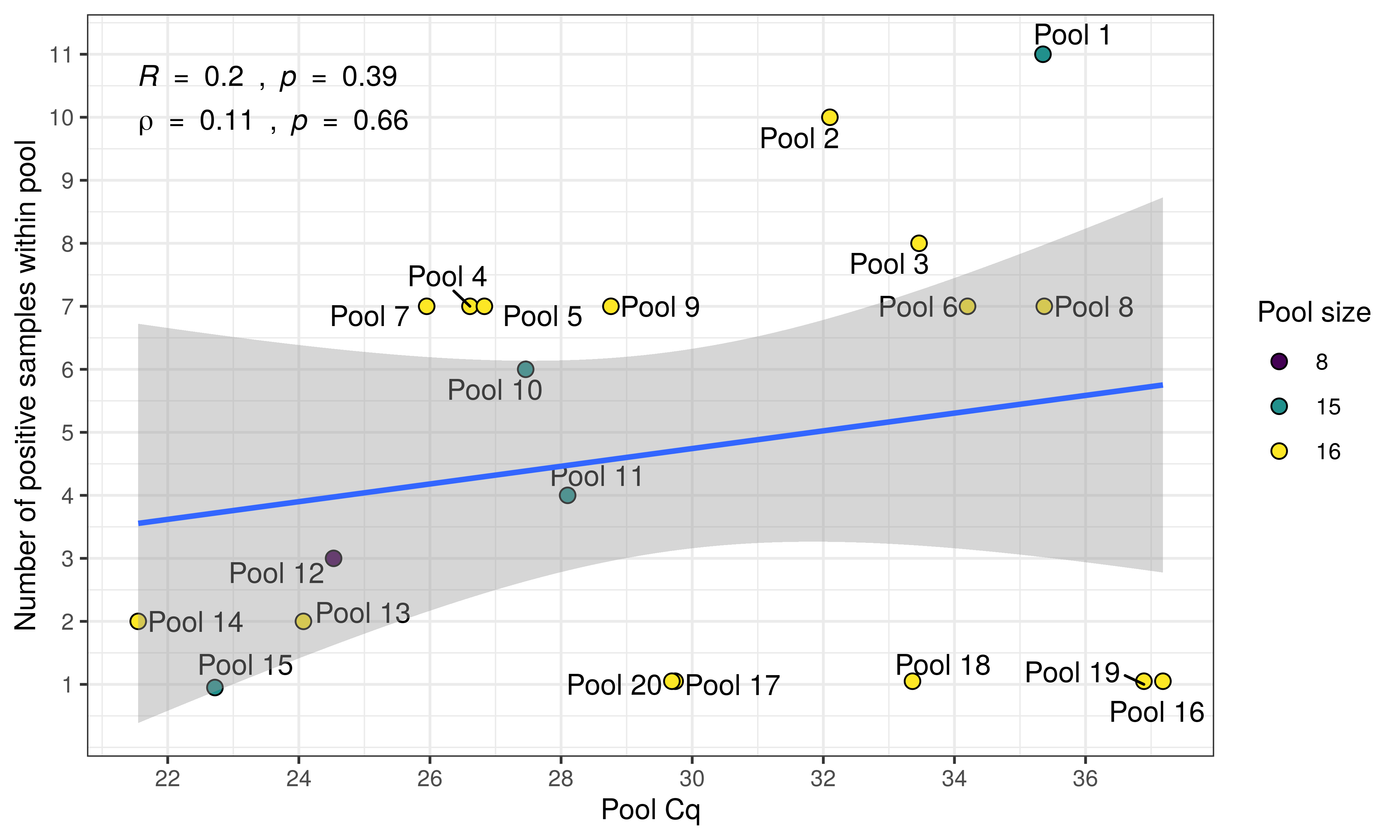

Supplement: S1 Data — (GZ) [file pone.0246544.s001.gz › output/img/cor_cq_pool_n_pos.png]

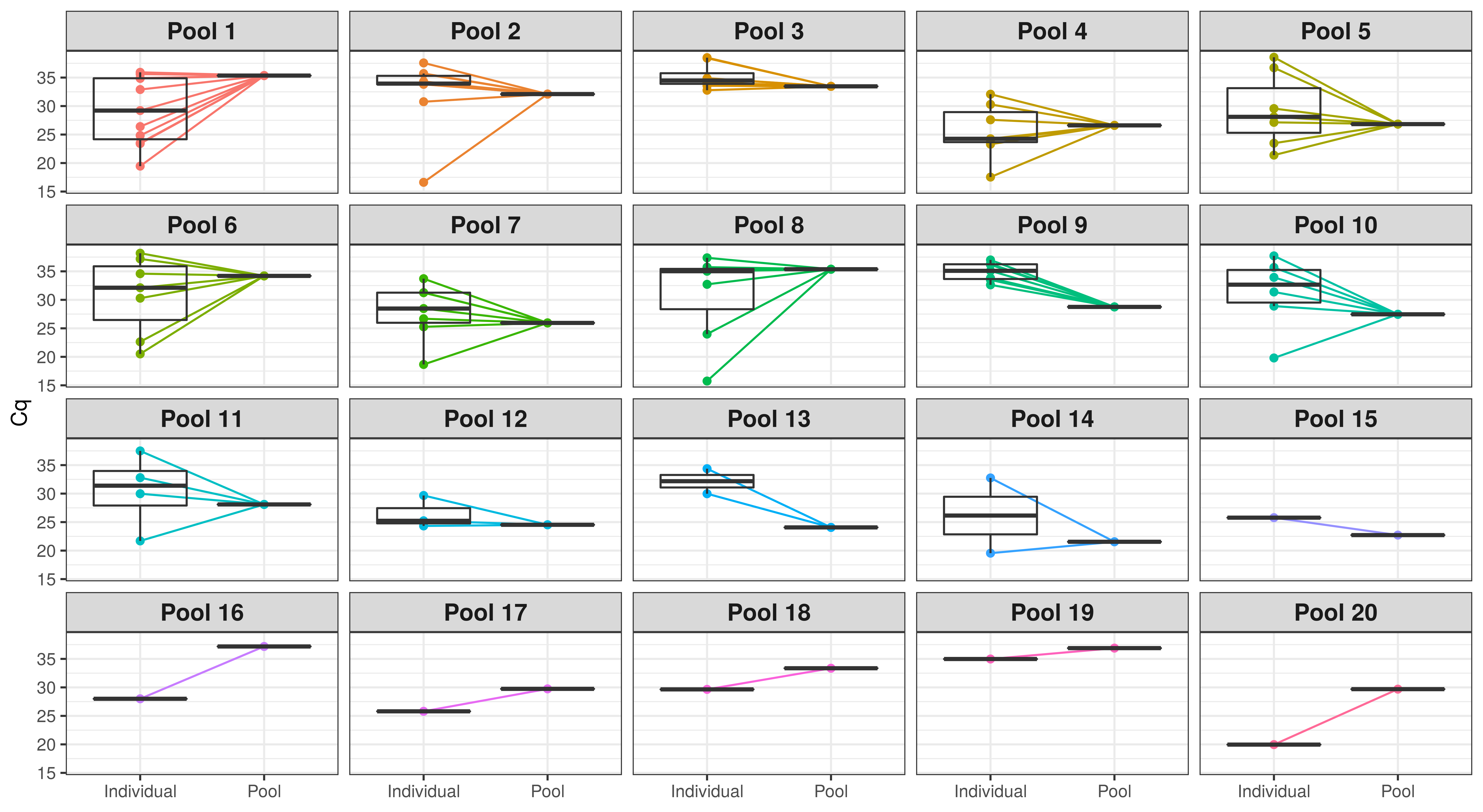

Supplement: S1 Data — (GZ) [file pone.0246544.s001.gz › output/img/pool_vs_indiv_cq_val.png]

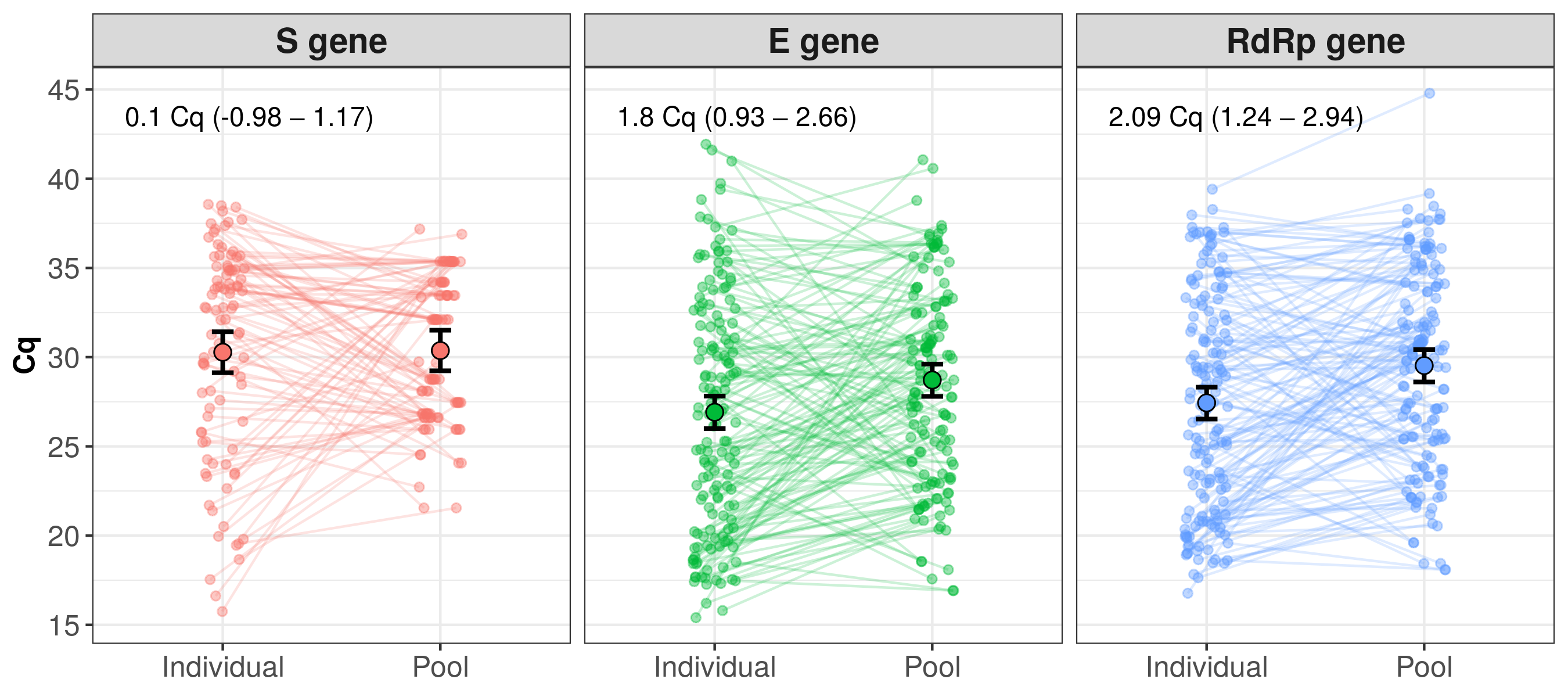

Supplement: S1 Data — (GZ) [file pone.0246544.s001.gz › output/img/cq_differences.png]

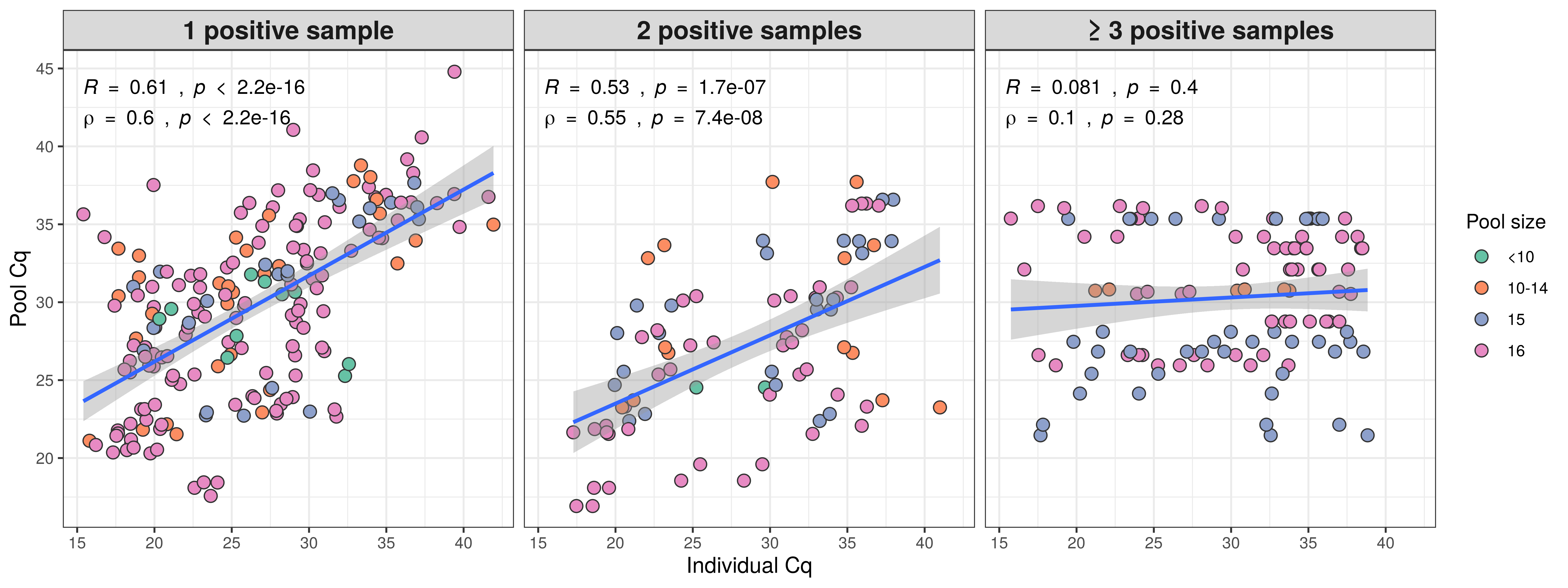

Supplement: S1 Data — (GZ) [file pone.0246544.s001.gz › output/img/corr_indiv_pool_cq.png]

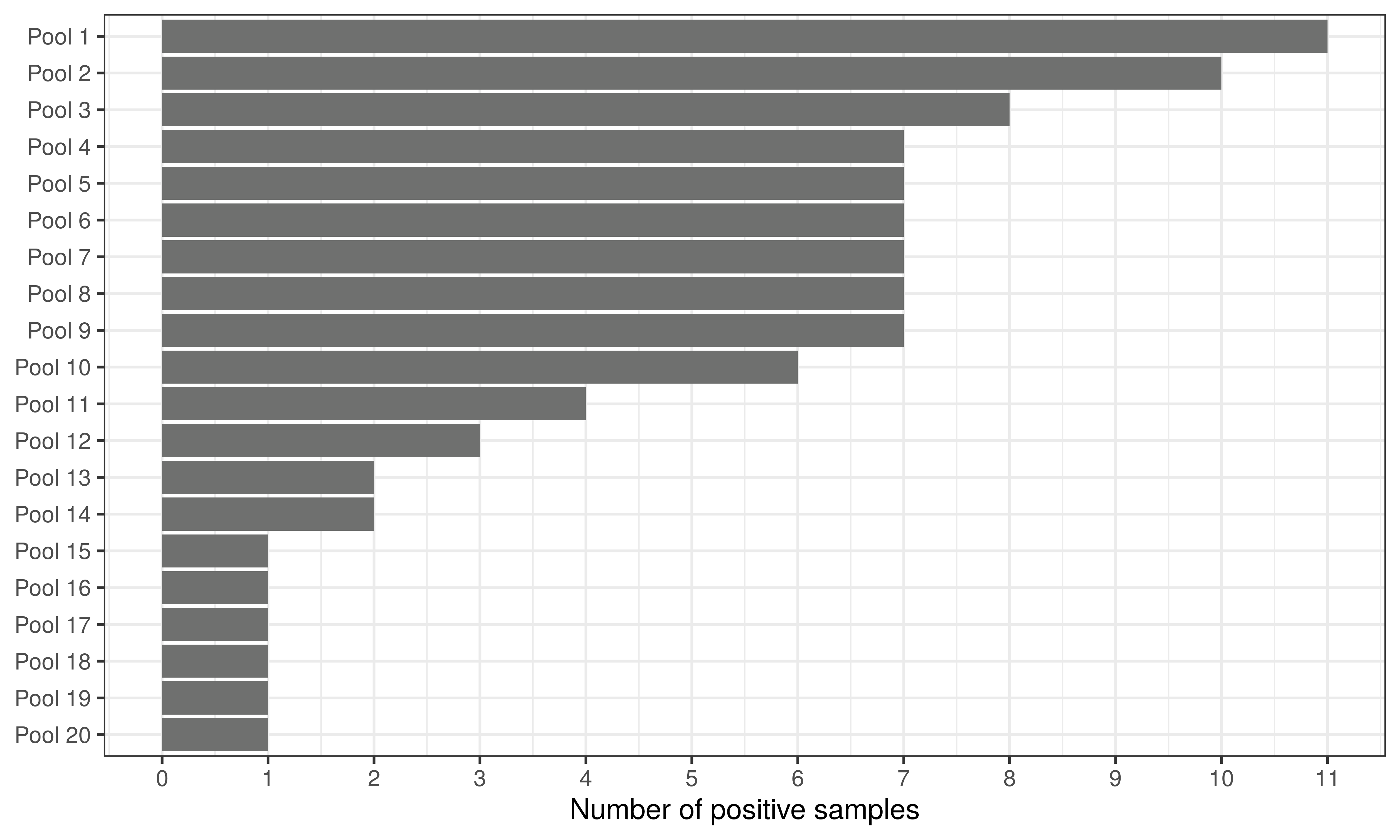

Supplement: S1 Data — (GZ) [file pone.0246544.s001.gz › output/img/positive_samples_per_pool_validation.png]
