# Supplementary material for: Swab pooling: A new method for large-scale RT-qPCR screening of SARS-CoV-2 avoiding sample dilution
Source: PLoS One. 2021 Feb 4;16(2):e0246544. doi: 10.1371/journal.pone.0246544 (PMC7861376; doi:10.1371/journal.pone.0246544)
Supplement: Attachment — Submitted filename: Response to Reviewers.docx [file pone.0246544.s002.docx]

Swab pooling: a new method for large-scale RT-qPCR screening of SARS-CoV-2 avoiding sample dilution (PONE-D-20-32256R1)

Rebuttal letter:

1. **The reason for using amplification cycles (Ct) of 40 or above is not clear.**

We have modified the text accordingly to clarify this point. We included more detailed information to consider a sample positive for SARS-CoV-2, which states as follows:

“For pool samples, detecting at least one gene with Cq value lower than 40 was enough for pool opening and subsequent individual testing; for individual samples, detecting both genes with Cq lower than 40 was required to consider it positive for SARS-CoV-2".

This was added to the methods section in lines 121-127.

Three individual samples had divergent results (one gene with Cq lower than 40 and another with Cq slightly higher than 40), but it was not possible to collect new samples. For precaution, the patients were treated as positive and, hence, their results were kept in the analysis. This was detailed in the rewritten lines 203-211.

2. **There is no comparison using COV2 antigen detection.**

We understand the interest in antigen tests due to the benefits of point-of-care diagnosis. However, comparisons involving antigen testing go beyond the scope of this work for several reasons:

a. **Antigen testing has lower sensitivity than RT-qPCR**: while antigen testing has become popular, it is recommended that negative results should be confirmed by RT-qPCR due to inferior sensitivity (1). Even though it is suggested that antigen tests are sensitive enough to detect active infection only, there is substantial uncertainty regarding its actual sensitivity, with reported values ranging from 80% to 98% under varying validation conditions (2-4). Most works assessing antigen tests used RT-qPCR as a unique reference, as we do here. Future works comparing multiple methods should be useful, but beyond our current operational capacity and scope.
b. **Antigen testing and swab pooling target different populations:** while swab pooling, as any other pooling strategy, targets low prevalence populations, antigen testing has been recommended to target symptomatic individuals (1,4). In fact, a recent assessment comparing antigen testing to standard RT-qPCR found a sensitivity of 80% (32 of 40) among symptomatic individuals but of only 41% (7 of 17) for asymptomatic individuals (3). Even though this matter certainly requires further investigation, there is not enough evidence to support the widespread adoption of rapid antigen tests as a substitute of molecular testing for the screening of asymptomatic people and other low prevalence populations. Both the FDA and Brazil’s National Health Surveillance Agency (ANVISA) recommend that negative antigen test results should be subsequently confirmed by RT-qPCR, which may further delay the process of ruling out infection (1,4). The use of antigen tests for screening is suggested only if molecular testing is not feasible or if turnaround times are too long.
c. **Our aim is solely to propose a reliable and efficient alternative for large-scale screening in low prevalence populations:** massive molecular testing for SARS-CoV-2 places logistic, operational, and financial challenges. The more alternatives available the higher our ability to address issues due to insufficient testing. Here, our proposal improves on sample pooling, a strategy successfully implemented worldwide (5-6).

Finally, works addressing different variations of pool testing continue to be published without any comparison to antigen testing (5-6). The value of our contribution lies in the improvement over the two main drawbacks from sample pooling: sample dilution and increased laboratory workload. Our method has allowed reliable screening of over 20 thousand individuals who would not have been tested by either individual RT-qPCR or antigen testing.

3. **Several statements do not seem appropriate i.e. the beginning of the discussion section should include the fact that the  extensive COV2 testing is essential for monitoring viral infection in the human population and a prerequisite for investigating how the virus spreads.**

While we are not sure which statements the editor refers to, we have reviewed our discussion section and included the suggested observations (lines 205-206), in addition to minor modifications along the manuscript.

REFERENCES

1. FDA. 2020. A Closer Look at Coronavirus Disease 2019 (COVID-19) Diagnostic Testing. Available at: <https://www.fda.gov/media/143737/download>. Accessed: 15/01/2021.
2. <https://doi.org/10.1016/S0140-6736(20)32635-0>
3. <http://dx.doi.org/10.15585/mmwr.mm695152a3>
4. ANVISA. 2021. NOTA TÉCNICA Nº 7/2021. Available at: <https://www.gov.br/anvisa/pt-br/centraisdeconteudo/publicacoes/servicosdesaude/notas-tecnicas/nota-tecnica-no-7-de-2021.pdf>. Accessed: 15/01/2021.
5. <https://www.thelancet.com/journals/laninf/article/PIIS1473-3099(20)30362-5/fulltext>

<https://www.nature.com/articles/s41586-020-2885-5>
